# The Present State and Prospective Trajectory of External Breast Prosthesis (EBP): A Global Overview

**DOI:** 10.7759/cureus.70685

**Published:** 2024-10-02

**Authors:** Lystra Deshong, Amalia Hosein, Kristy Samaroo

**Affiliations:** 1 Biomedical Engineering, The University of Trinidad & Tobago, Port of Spain, TTO

**Keywords:** breast cancer, external breast prosthesis, mastectomy, quality-of-life, women

## Abstract

This study intends to examine and describe the current and future status of the utilization of external breast prostheses (EBP) amongst global women diagnosed with breast cancer who have had a mastectomy. To avoid breast reconstruction, many women choose external breast prostheses (EBP) for varied reasons. This study included literature from ScienceDirect and PubMed to select relevant global articles from the year 2011 to the year 2023. The terms “external breast prosthesis breast cancer” were used as a search title for abstracts and keywords to source articles from both databases. Similar and related terms were used concurrently for searches conducted in ScienceDirect and PubMed. For this mixed-method review, qualitative data gave way to an in-depth exploration of the phenomenon, while the presentation of quantitative data demonstrated the tabulation of global publications on EBP. All studies focused on EBP use among post-operative women. A total of 22 journal articles were selected for this review. From the data six (6) themes were identified *viz.*; patient satisfaction, comfort, quality of life, knowledge about external breast prosthesis, cost, and material. Our findings showed that the literature had fifteen (68%) of the publications on patient satisfaction, sixteen (73 %) on comfort, twenty (91%) on quality of life, and fifteen (67%) on material. From the published literature, there was an observed dearth of information on knowledge and cost with only eleven (50%) and eight (36%) articles respectively, which indicates limited access to pertinent information for both healthcare providers and patients. The results suggest that more research is needed to provide the best quality information and approaches when looking at EBP for female breast cancer care and post-operative rehabilitation.

## Introduction and background

Globally, 7.8 million women were diagnosed with breast cancer at the end of 2020, as reported by the World Health Organization [1]. Recently, researchers have increased interest in breast cancer, since empirical data shows that this type of cancer is most common among other types of cancer across the globe, which mainly target females [2]. According to Samaroo K et al., 2021 [3], when reviewing mortality, breast cancer in women accounts for most cancer-related deaths among females around the world. As such, this dreaded disease has become a serious public health concern and the latest global trend [4]. Previous studies focused primarily on the trend in older women, without paying close attention to the younger female population [5], hence, misrepresenting this particular stratum, without considering the effects of breast cancer in its entirety.

Although a breast cancer diagnosis substantially impacts women at any age, younger women fare worse. This concept should not go unnoticed as younger women symbolize the strength of a country’s workforce and conduits for procreation. Being diagnosed <45 years old places these women in a bind to experience challenges in familial relationships and other psychosocial dilemmas [6]. According to the authors, younger women’s lives become more complex when breast cancer strikes at a stage in life whilst juggling careers, actively being employed, or trying to balance family life.

Cancer care involves different modalities of treatment, such as breast conservation surgery (lumpectomy), mastectomy, chemotherapy, radiation, and hormone therapy, among others, for management efficacy [7]. Jetha et al., (2017) [7], regard mastectomy as a vital modality treatment since this surgical intervention at times is necessary to save lives. One study [8] encourages women to have themselves screened early and regularly, given that mastectomy was found to be much more prevalent than breast conservation surgery when breast cancer is diagnosed late-stage. 

Based on scientific evidence, mastectomy causes serious psychological repercussions among post-operative women [9]. Post-operative dilemmas contribute to depressive behaviours including, but not limited to; isolation, low self-esteem, lack of intimacy, and avoidance of going out publicly, which gives way to a reduced quality of life [10]. To deal with breast loss, mastectomized women seek breast restoration alternatives to help them feel better about themselves by way of either breast reconstruction or external breast prostheses (EBP) [11].

**Figure 1:**
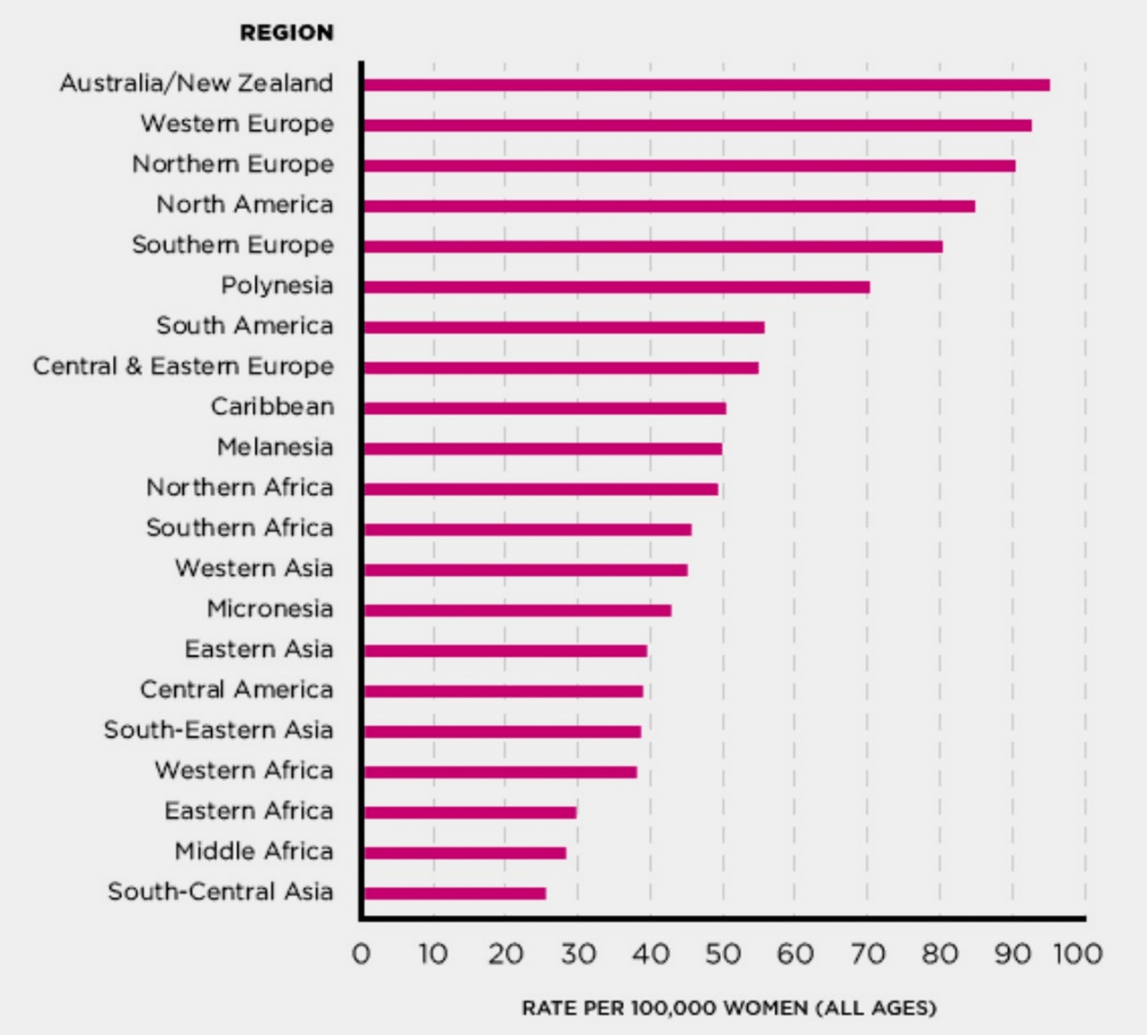
Global rates of female breast cancer. Source: © International Agency for Research on Cancer (IARC) and World Health Organization (WHO) [12] - Open Access Article.

Figure 1 highlights breast cancer incidences around the globe. According to GLOBOCAN, (2012) [12], statistically out of 100,000 women, Australia/New Zealand (99.5 per 100,000), Western Europe (99.2 per 100,000), North Europe (90 per 100,000) and North America (88.5 per 100,000) are seen to have the highest incidences of breast cancer. Conversely, Middle Africa (29 per 100,000) and South-Central Asia (26 per 100,000) are among the lowest countries. What is quite alarming is that although the Caribbean has a low population, relative to other countries, it is noted that the Caribbean region accounts for 50 per 100,000 women which is way too high per capita. Interestingly, the incidence in the Caribbean is comparable to that of the African continent.

**Figure 2:**
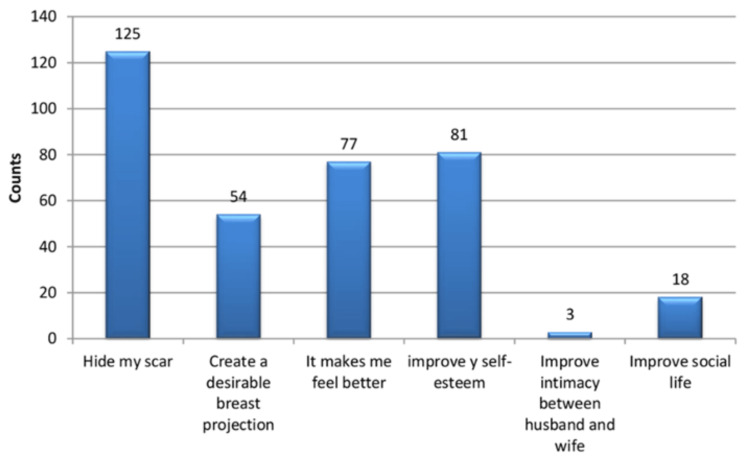
Number of women who have had a mastectomy and chose to use external breast prosthesis in Hong Kong. Source: Leung (2017) [14]. Published with permission from the author.

Many breast cancer patients forego breast reconstruction surgery due to factors such as age, the notion of recurrence, fear of being subjected to further surgeries, and fear of failed reconstruction are concerns linked to the surgical procedure [13]. For such individuals, external breast prosthesis (EBP) becomes the most appropriate choice, as it provides a vital solution to regain body image. Therefore, accentuating a positive effect, according to Leung [14] when it comes to hiding scars, improving appearance, bettering intimate relationships, creating better breast projection, heightening self-esteem, and having a better social life as seen below (Figure 2).

Over 80% of women in Western countries choose to wear external breast prostheses (EBP) post-surgery [15]. As such, this study sets out to review the existing body of research on EBP, to present data found, hoping to contribute a deeper understanding of the advancement of EBP from then to now, since EBP plays a critical role in maintaining post-mastectomy women’s self-esteem and self-confidence. Consequently, this enables them to continue going about their lives, while engaging in normal daily activities according to Qiu et al. [15].

## Review

Methods

Literature Search

**Figure 3:**
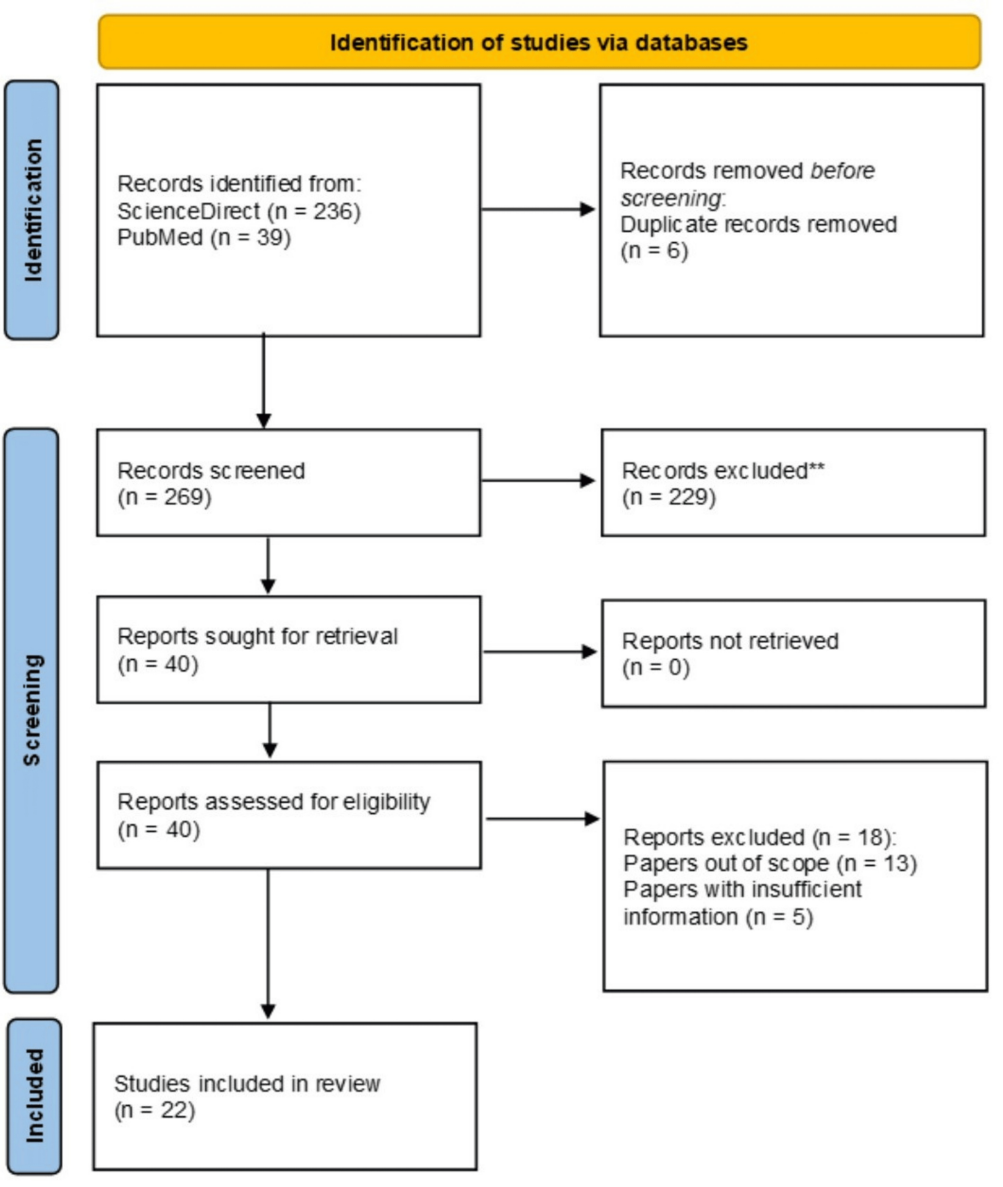
PRISMA flowchart of selection and screening of studies The figure was created using the PRISMA 2020 flow diagram template [16]. PRISMA: Preferred Reporting Items for Systematic Reviews and Meta-Analyses

A systematic literature search was conducted for articles that assessed the utilization of “external breast prosthesis (EBP)” for mastectomy women across the globe in Figure 3, following the PRISMA guidelines [16]. The search for this review was conducted between June 2023 and February 2024 using ScienceDirect and PubMed databases. The search extensively reviewed oncological and medical journal articles on EBP in English, published from 2011 to 2023. The search term “external breast prosthesis breast cancer” was used concurrently with similar and related terms in both ScienceDirect and PubMed to broaden the search.

Inclusion Criteria, Screening, and Data Extraction

Duplicate records were removed and results were organized and examined individually. Titles, keywords, and abstracts were subsequently examined to identify articles that assessed the use of external breast prostheses (EBP) among global post-mastectomy women. Through searching, once the identified title and abstracts were ambiguous, full-text articles were then retrieved and examined to review the full-text eligibility. The inclusion criteria were; (i) peer-reviewed medical and oncological journal articles on external breast prosthesis as a post-surgical breast form versus information on breast reconstruction, (ii) EBP current and future trends, (iii) publications in English between the period of 2011 and 2023 and (iv) breast cancer women who had a mastectomy as a specific modality of oncological treatment. 

Articles were excluded based on the following; (i) data from books, editorials, thesis papers, and unambiguous abstracts with insufficient information, (ii) literature that mainly focused on breast reconstruction as a type of breast form (iii) not published in English, (iv) published earlier than 2011 and (v) out of scope information such as different modalities of breast cancer treatment other than mastectomy such as radiation and/or chemotherapy treatment. For each article, the following were extracted; gender and type of cancer (female breast cancer), current and future use of external breast prosthesis (EBP), the author’s name and date of the publication, the different global countries were grouped and six emerging themes were discovered viz; patient satisfaction, comfort, quality of life, knowledge on EBP, cost and material.

Results

Figure 3 highlights the initial mapping steps in accordance with PRISMA guidelines [16], which aids in identifying relevant papers used for this systematic review. These papers were published between 2011 and 2023, covering a 12-year period. 269 records were screened from ScienceDirect and PubMed databases for potential inclusion criteria. After screening, a total of 22 research articles presented results on the present state and future trajectory of external breast prosthesis (EBP) use among global breast cancer women were found to be eligible. As per global publications, Poland (n = 5) had the most articles, followed by China (n = 4), Australia, Germany, India, Brazil, and Mexico (n = 2) respectively with Spain, Canada, Egypt, and Turkey representing (n = 1). Six overarching themes (patient satisfaction, comfort, quality of life, knowledge about EBP, cost, and material) were identified to illustrate factors that influence EBP use for female post-surgical breast cancer patients.

Thematic Analysis

**Figure 4:**
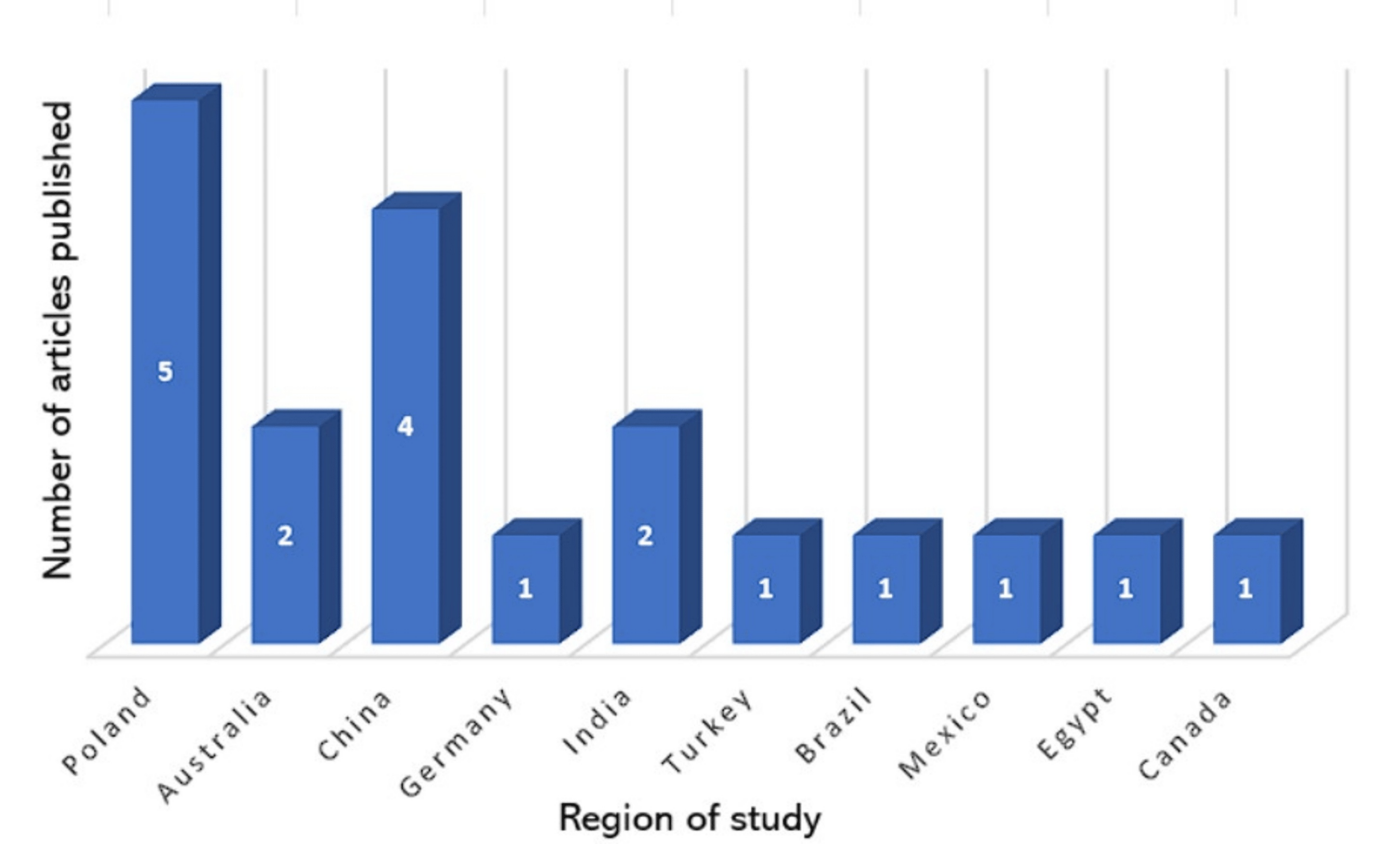
Number of articles per country used in this review Image Credit: Lystra Deshong, 2024

From the literature review, the following themes emerged, as summarized in Table 1. Table 1 shows that articles with inclusion criteria focused on (1) patient satisfaction, (2) comfort, (3) quality of life, (4) knowledge about EBPs, (5) cost, and (6) material. Notably, only eleven (50%) of the articles covered knowledge about external breast prostheses (EBP), and as reviewed, eight (36%) covered cost. The majority of studies done on EBP (Figure 4) were seen in Poland, n=5 (23%). Turkey, Egypt, Spain, and Canada had the lowest publication records, n=1 (4.5%) each. There was no record of literature for the Caribbean on EBP.

**Table 1:** Themes identified from the 21 publications within the inclusion criteria.

Themes	Number of Articles	Percentage
Patient Satisfaction	15/21	71%
Comfort	15/21	71%
Quality of Life (QOL) Post-Surgery	19/21	90%
Knowledge About External Breast Prosthesis	11/21	52%
Cost	7/21	33%
Material	14/21	67%

Patient Satisfaction

According to this review, fifteen (n=15) articles, 68% of the literature, investigated patient satisfaction in women wearing external breast prostheses (Table 1). Findings suggested issues such as body image and self-esteem are highly pivotal when reviewing mastectomy [17]. For this cause, postmastectomy women who refused reconstruction surgery said they were satisfied with EBP as a restorative breast form to help improve their self-esteem [18]. Jetha et al., [7] study claimed that women’s satisfaction increases when EBPs are worn continuously, as opposed to wearing them infrequently. Wearers allowed to choose EBP themselves expressed more satisfaction than those who had to subscribe to what was offered to them [19] [20]. A previous study by Kubon et al., (2012) [21], pleaded with manufacturers to improve their products, thus fostering better satisfaction and consistency when it comes to proper fitting [21]. 

Current data highlights the evolution of EBPs as manufacturers around the globe are claiming that women of all ages are now able to find EBPs that approximate the ‘‘shape and drape’’ of their existing breasts [22]. Crompvoets (2012) [22] showed that up-to-date EBPs are uniquely made to satisfy today’s women, regardless of their skin tones, breast sizes, and shapes, and also cater to those without nipples and areolas. Recent literature also focused on newly designed heat-reduction EBPs [11] [23]. According to the authors, users of such type of prosthesis exhibited lower core temperature responses than those wearing the conventional mastectomy bra. Consequently, users said they felt as though the temperature-controlled bra integrated well with their bodies and as such, they were satisfied using the product. Additionally, novel development with 3D technology has emerged as the future evolution for breast prostheses and breast cancer treatment. Patients surveyed in a study by Montejo et al. (2020) [24] said they were satisfied with EBP made by 3-D printing, as it mimics the realness of a natural breast.

In this review patients’ dissatisfaction was not downplayed, since several studies highlighted weight as a contributing factor to patient dissatisfaction. In Poland, 24% of mastectomy recipients reported dissatisfaction due to the weight of some prostheses [25]. Comparative studies agree that standard-type EBP causes dissatisfaction due to its heaviness [21, 26-27]. For this cause, prostheses are produced in a wide range of shapes, sizes, and skin tones other than standard-type EBP that are designed to move, feel, and weigh as similar to a mutual breast as possible [28]. To ease EBP weight, 19% of women in India refused to use commercial EBPs instead satisfaction was reported through the use of homemade mastectomy bras, which were sewn with padded cotton inside the bra cups to configure well-fitting and supportive bras [29]. Brazilian single young women also voiced dissatisfaction with EBP and contemplated reconstructive surgery. However, their older counterparts, said though they experienced inconsistencies with; stitches, pains, pulling, dormancy, and phantom limbs, they were still satisfied using EBPs [30].

In concluding the theme of patient satisfaction, one must bear in mind that many mastectomized women forego breast reconstruction surgery for various reasons. According to Hojan (2020) [19], EBP acts as a key tool to bring satisfaction to postoperative women. For this review, 71% of the literature tapped into satisfaction, without downplaying patients' dissatisfaction. Results showed that factors such as weight and the inability to choose EBPs triggered dissatisfaction. Emergent studies confirm science is revolving around both 3-D technology and temperature-controlled EBPs to address overall patient satisfaction and the lack thereof.

Comfort

Comfort was highlighted in sixteen (n=16) articles, 73% of the literature. According to Kubon et al., (2012) and McGhee et al., (2020) [21, 27], comfort influences prosthetic breast use, the authors claimed that most women in their studies said they felt comfortable using EBPs. Accordingly, lightweight EBP provides better comfort since it places less pressure on women's bra straps as opposed to wearing standard-weight EBP [20]. The view of lightweight EBP and comfort was supported by Polish women, who said they felt more comfortable wearing custom-made prostheses [19]. A study conducted by Manikowska et al., (2019) [31] revealed that wearing EBP on the operated breast of equal weight to the healthy breast provided better balance and comfort. The result from Manikowska et al., (2019) [31], finding reflects that of Koralewska et al., (2023) [13], which confirms that EBPs significantly impact the way mastectomized women's bodies lean while giving them postural stability which in turn fosters comfort.

Extensive research by Qiu et al., (2020) and Leung et al., (2021) [11, 23], investigated heat-reduction EBP made with temperature control technology. Studies revealed that this type of EBP absorbs body heat which in turn, keeps the chest cool and comfortable during hot summer days. Some participant said they felt more comfortable wearing cotton EBPs since they found silicon EBPs were too heavy, and vice versa those who wore silicon voiced their discomfort when wearing cotton as they said cotton was too light in weight and did not provide chest symmetry [7, 30]. In a similar study, Liang and Xu (2015) [26] surveyed patients in China and found that silicone was too hot for the summer and too cold for the winter, hence they said such issues caused discomfort. 17% of persons indicated issues of discomfort when looking at the weight of EBP [25]. Patients surveyed agreed that the new (3D) prosthesis was more comfortable, and lighter, and made it easier for them to choose their wardrobe [24].

Overall, external breast prosthesis is reviewed as a product geared to offer confidence, balance security, and most importantly comfort [22]. Issues such as weight, temperature, and standard-weight material were highlighted as some of the irregularities when examining comfort. Based on current studies, science keeps evolving with new 3D prostheses and ergonomic bras to address the aforementioned problems. While progress continues, the hope of attaining comfort remains in the hearts of breast cancer sufferers who had mastectomies and refused esthetic surgery.

Quality of Life

A focus on the quality of life (QOL) of EBP users received the highest score in the literature with twenty (n=20) articles, (91%). Studies conducted by McGhee et al., (2020) [27], found that lightweight EBP places significantly less pressure on participants' bra straps during daily activities, which in turn helps to create a positive effect effective on the physical well-being of women post-mastectomy. A study by Hojan, (2020) [19], noted that with EBPs, participants were able to exercise and move around easier, and the weight of the external device did not affect them. Detailed studies on the influence of body posture affirmed that EBP helps to adjust postural instability, and offers better movement for physical activities, hence fostering a better quality of life [7, 13, 25, 32]. A comparative study by Qiu et al., (2020) [11], agrees that EBP use prevents long-term complications, including dropped shoulders syndrome, and asymmetry, and ultimately improves the QOL of patients.

After breast loss, the provision of an appropriate External Breast Prosthesis (EBP) helps women to recover; emotionally, socially, and physically [28]. The components of self-confidence, body image, and social functioning were explored as to the usefulness of EBP and quality of life; it was reported that EBP offered users a sense of normalcy, women < 50 exhibited fewer body image issues, and regained self-confidence which helped to increase physical and social activities [17, 20, 29, 30]. According to Crompvoets (2012) [22], participants in their study say ‘EBP offers them full freedom of movement and complete confidence to pursue activities like tennis, golf, jogging, and swimming, offering them a new quality of life.’

In terms of new designs, Cruz et al., (2018) [33], propose that 3D EBP is designed to perform similarly to the real breast regarding structure, symmetry, consistency, contour smoothness, mobility, and a sensation that is realistic to the touch. According to the researchers, a prosthesis with such features would contribute significantly to the patient’s physical rehabilitation and social integration. Also, examining recent products on the market, Leung et al. (2021) [23] observed that ventilation holes in the heat reduction bras encouraged more airflow, which expedites heat dissipation and sweat evaporation. As such participants said they were better able to cope with daily activities. According to Montejo et al., (2020) [24], researchers must work effortlessly for the progression of EBPs, as proper designs will help users to adapt better to EBPs, which in turn can increase patients' morphology and improve their lifestyle quality after mastectomy surgery.

Literature on quality of life was explored comprehensively. Findings revealed that EBP has a positive impact on its users. Previously, post-operative women viewed EBP negatively, but through educative and marketing strategies their mindsets were altered. Today, women around the globe report that using EBP helps to maintain their physical appearance, self-esteem, confidence femininity, normality, and body image, alternately improving their quality of life [15, 18]. Literature on EBP and quality of life in China is limited; as Liang and Xu (2015) [26] showed, there was no difference in the quality of life between Chinese patients whether they chose to wear an EBP or not after mastectomy.

Knowledge About External Breast Prosthesis

In examining patients' knowledge, this section presents information from eleven (n=11) articles, 50% of the literature. In China, Qiu et al. (2021) [15] said, “Presently, there is a lack of information about the use of EBP among Chinese patients, and medical professionals' knowledge concerning EBP is also poor.” Similarly, Liang and Xu (2015) [26] concur medical professionals, especially specialist breast nurses, should be more forthcoming with comprehensive and educational information about EBP use so Chinese patients can choose aptly, and better navigate their physical or mental challenges. Data gathered by Jetha et al. (2017) [7] and Ramu et al. (2015) [29] highlighted the plea of participants in India, as post-operative women requested EBP information inclusive of prosthesis types, cost, and shop locations. Participants said such information should be given to patients and their families in aid of fostering efficacy when it comes to rehabilitation. Ramu et al. (2015) [29] further expounded that education and age strongly affected EBP use. Data showed most of the well-educated patients used EBP while women in rural areas refused commercial bras and chose homemade bras instead.

Data from several other global studies identified the need for professional breast prosthesis information. Wiedemann and Schnepp (2017) [20] confirm there is a lack of information and choice in prosthesis fitting is a common problem in Germany. Hessin et al., (2021) [18], suggested global surgeons placed more emphasis on providing information on breast reconstruction rather than EBPs. In their research, the authors found instructional guidelines were extremely effective in improving knowledge, practice, and self-esteem regarding external breast prostheses among post-mastectomy women. Conversely, in Australia, some patients said that they were informed about EBP by their oncology nurse while others said they found out on their own [27]. Whereas Compvoets (2012) [22] provided proper information to participants via a 15-page booklet on strategies on how to cope with breast cancer and decision-making tips when choosing EBP. Kubon et al. (2012) [21] show participants who were part of their trial reported that access to EBP information was very satisfactory. Also, the Borghesan et al. (2014) [30] study calls for health education activities to be extended to the entire process of treatment of the disease, including contributing to the orientation and compliance with the use of EBP and the quality of life. A suggestion made by Hojan (2020) [19] is that clinical nurse instructors should plan in-service sessions to educate clinical staff about the importance of EBP for the rehabilitation of mastectomy patients, especially in psychological aspects.

A considerable amount of EBP literature was published on patients’ satisfaction, comfort, and quality of life, but why so little knowledge of the product? Although participants in Australia reported they received satisfactory information, many participants said professionals need to be more vigilant in educating them about the product. Health practitioners must bear in mind that lack of knowledge is very critical to women’s health, which will ultimately lead to poor rehabilitation. Consequently, as seen by Ramu et al. (2015) [29] suburban women in India were placed at a greater disadvantage than their urban counterparts with EBP usage. With little or no access to information, some post-operative women turned to the use of hand-made bras stuffed with rice or cotton. Current research needs to be done, thus providing information to fill this gap. 

Cost

A mere eight (n=8) articles, 36% of the literature, covered EBP costs. Participants in a study by Hojan (2020) [19] say, “The high cost of EBP and affordability was the main concern for breast cancer women in Poland after mastectomy.” An economic plea was made by Ramu et al. (2015) [29] for commercial EBPs to be made more affordable, especially for persons from rural backgrounds with low economic status, thereby helping to boost their self-esteem as their suburban counterparts. Due to the lack of a general health insurance system in Pakistan, Jetha et al. (2017) [7] acknowledged that most people had to pay out-of-pocket for EBP services. Hence, participants highlighted cost as a serious issue in obtaining EBPs. Similarly, Liang and Xu (2015) [26] posed that the cost of prostheses was seen as a significant factor influencing the choice for utilization of the product. As such, Liang and Xu (2015) [26] said the existence of a government fund was considered as an option to eliminate the influence of cost, due to patients’ financial situation. Kubon et al. (2012) [21] claimed that both custom and conventional breast prostheses are 75% funded by the Canadian government, leaving the patient responsible for incurring only 25% of the cost.

To better inform participants about cost, a strategic advertisement by Crompvoets (2012) [22] provided information via a 15-page booklet for Australian women so that proper insight was granted on the availability, types, and cost of EBPs. An advanced study by Montejo et al. (2020) [24] assumes that the new 3D digitized EBP will be cost-effective. Participants from the study say they are "willing to pay out-of-pocket for the digitized bra as it proposes to be comfortable and attractive.”

As has been established, cost plays a major role in EBP accessibility. In the literature, it was reported that some governments fund EBP programs, regardless, some participants verbalized their challenges with the cost factor of EBP, therefore having to incur out-of-pocket expenses to access the product. However, newer technology promises that future EBP will be more satisfactory and cost-effective [24]. Hopefully, users can benefit economically.

Material

Fifteen (n=15) articles, 68% of findings, pointed to the different types of material used in EBPs. Investigators measured both lightweight EBPs (silicon/cotton) and standard-weight silicon EBPs to find out which of the EBPs felt more comfortable and fit better [11, 18-19, 26-27]. Another study found that most participants preferred lightweight EBPs [20]. However, for those who chose custom-made EBP, a custom-made silicone palette with matching colors was used [21]. Pakistani women who used silicone said the material was too heavy and preferred cotton or foam types EBPs [7]. Ramu et al. (2015) [29] reported: “Silicone prosthesis was used by 22% of women, 44% women used cloth or cotton padded bra cups and others used other types of prostheses, as women in the rural areas who refused commercial EBPs were said to be stuffing their bras with cloths while some wore handmade cotton bras.”

An updated investigation of ergonomic EBPs showed that a polyurethane type of material was used as a design for heat reduction [23]. The computational design via 3D model uses the physical properties of an elastomeric material as a new way to design EBPs to mimic the realness of a healthy breast [23]. Elastomeric has a high coefficient of friction, softness, and flexibility. This type of material is completely odorless and does not produce toxic gases [34]. Added to this, the 3D technology approach is fast and offers a customizable material that is low-cost, by using a flexible approach when producing EBP devices [35]. In agreement with Ramirez et al.'s (2020) [34] biometric study, Montejo et al.'s (2020) [24] research says, “3D main printing material used was the thermoplastic elastomeric filament (TPU) with a polyurethane base along with PVA (polyvinyl acetate) water-soluble filament as a layer of supportive material.”

Concerning material, previous manufacturers advertised EBP bras made from foam and standard and light silicon in various sizes, weights, and shapes tailored for use during different activities [22]. However, modern-day research points to 3D designs as imminent. The research aims to consider the potential psychological and physical effects that mastectomy surgery bears on postoperative women. Thus, innovating new biometric technology to mimic the realness of a breast.

Table 2 summarizes the articles used for this review by global territory and provides context on the themes that were discussed in each.

**Table 2:** Information covered for each review article according to global territories. √ means the theme was discussed in the article. x means the theme was not discussed in the article.

First Author	Country	Patient Satisfaction	Comfort	Quality of Life	Knowledge on EBP	Cost	Material
Mc Ghee et al., 2020 [27]	Australia	√	√	√	√	x	√
Crompvoets et al., 2012 [22]	Australia	√	√	√	√	√	√
Leme et al., 2023 [35]	Brazil	×	√	√	×	√	√
Borghesan et al., 2014 [30]	Brazil	√	√	√	√	x	x
Qiu et al., 2021 [15]	China	x	x	x	√	x	x
Qiu et al., 2020 [11]	China	√	√	√	√	x	√
Liang et al., 2015 [26]	China	√	√	√	√	√	√
Hessin et al., 2021 [18]	Egypt	√	x	√	√	x	√
Wiedemann et al., 2017 [20]	Germany	√	√	√	√	x	√
Leung et al., 2021 [23]	Hong Kong, China	√	√	√	X	x	√
Jetha et al., 2017 [7]	India	√	√	√	√	√	√
Ramu et al., 2015 [29]	India	√	√	√	√	√	√
Ramírez et al., 2020 [34]	Mexico	x	x	x	X	x	√
Cruz et al., 2018 [33]	Mexico	x	x	√	X	x	x
Koralewska et al., 2023 [13]	Poland	x	√	√	X	x	√
Hojan et al., 2020 [19]	Poland	√	√	√	X	√	√
Manikowska et al., 2019 [31]	Poland	x	√	√	X	x	X
Hojan et al., 2017 [32]	Poland	x	x	√	X	x	X
Hojan et al., 2016 [25]	Poland	√	√	√	X	x	X
Montejo et al., 2020 [24]	Spain	√	√	√	X	√	√
Kubon et al., 2012 [21]	Toronto, Canada	√	√	√	√	√	√
Akkaya et al., 2011 [17]	Turkey	√	x	√	X	x	X

## Conclusions

External breast prostheses significantly enhance the body image and self-esteem of post-mastectomy women, offering an improved quality of life. Advances in 3D technology have led to ergonomic designs that better mimic natural breasts, increasing comfort and satisfaction. Healthcare providers should inform women about these options to aid in their rehabilitation. It is important to address access disparities for rural women and consider financial assistance policies for EBPs. Additionally, research on EBP usage in the Caribbean is needed due to the high incidence of female breast cancer in the region.
